# “This Student Needs to Stay Back”: To What Degree Would Instructors Rely on the Recommendation of Learning Analytics?

**DOI:** 10.1007/s42979-022-01137-6

**Published:** 2022-04-29

**Authors:** Linda Mai, Alina Köchling, Marius Claus Wehner

**Affiliations:** Heinrich-Heine-University Düsseldorf, Faculty of Business Administration and Economics, Universitätsstr. 1, 40225 Düsseldorf, Germany

**Keywords:** Learning analytics, Recommendation, Experimental design, Adaptive choice-based experiment, Scenario-based study, Educational data

## Abstract

Learning analytics (LA) systems are becoming a new source of advice for instructors. Using LA provides new insights into learning behaviours and occurring problems about learners. Educational platforms collect a wide range of data while learners use them, for example, time spent on the platform, passed exams, and completed tasks and provide recommendations in terms of predicted learning success based on LA. In turn, LA might increase efficiency and objectivity in the grading process. In this paper, we examine how instructors react to the platform’s automatic recommendations and to which extent they consider them when judging learners. Drawing on an adaptive choice-based experimental research design and a sample of 372 instructors, we analyze whether and to what degree instructors are influenced by the provided data and recommendations of an unknown LA system. In a follow-up study with 95 teachers, we describe the differences in the use of data between learners and the influence of early warning systems. All in all, we show the influence of automatic evaluation on teachers.

## Introduction

Due to the increasing digitization in educational institutions and the associated use of digital learning platforms [1], a vast amount of data is generated concerning the learning process, the learning progress, the learning outcome, and the learners themselves [2]. The COVID-19 pandemic may have accelerated this process [3]. Many platforms evaluate data automatically and additionally provide these for instructors to address the problem of differentiation [4]. Learning analytics (LA) is defined as a systematic analysis of large amounts of data about learners, instructors, and learning processes to increase the learning success and make teaching more effective and efficient [5]. Given the numerous opportunities of LA, the focus was rather on learners, their learning success and designing activities [2]; however, the platforms and LA might influence instructors as well.

Relying on the framework by Greller and Drachsler [5], instructors are involved as stakeholders when using LA. Consequently, they should not be overlooked when researching stakeholders. From an instructor’s perspective, platforms provide access to new information usually hidden in traditional learning contexts, such as learning behaviour and time spent with the offered materials online. This can improve the planning of teaching activities [6], but might influence the instructor’s judgment.

Judgment accuracy is the instructors’ ability to assess learners’ characteristics and adequately identify learning and task requirements [7]. When instructors need to assess, they can be biased. Instructors can be affected by ethnic and social backgrounds [8], expectations [9], halo effects [10], and other impacts that influence judgment accuracy [11]. Despite the growing use in practice, research about LA’s influence on instructors’ judgment is still limited. Therefore, these studies aim to examine to what extent instructors might be influenced in a setting with information and recommendations provided by LA.

We examine the influence of LA systems on instructors in two consecutive studies. The first study (Study 1) analyzes the influence of various information about learners on instructors’ evaluation provided by an unknown LA system. The second study (Study 2) focusses on the extent to which instructors' judgments change due to additional information provided by an LA system. In Study 1, we empirically analyze different evaluation criteria. The analysis relies on an adaptive choice-based conjoint analysis (ACBC) based on a sample of 372 instructors in Germany. Using a scenario-based experiment, we seek to identify the influence of different characteristics on taking platform information into account in Study 2. The analysis is based on another sample of 95 instructors from Germany. The contributions of these studies are both theoretically and practically relevant.

## Theoretical Background

### Learning Analytics

When using platforms in educational contexts, data about learners are generated, measured, collected, analyzed, and reported with the aim of improving learning an learning environments [12, 13]. This means a range of educational (meta)data is analyzed automatically to provide more information about learners. Information can be used to promote learners’ reflection, but they are also interesting for prediction systems of learners’ success [5]. The goal of LA is to analyze learners and their learning behaviour in such a way that learning practices can be individually adapted to the needs of the learners and thus become more effective [4]. LA can include machine learning methods to evaluate and monitor learning activities [14]. Although all stakeholders have an interest in data and learning success, Greller and Drachsler [5] distinguish between learners and instructors. Learners come up with data and gain feedback on their learning. Instructors receive data reports from the platform and act accordingly. That means they can adjust lessons and tasks to the learners’ requirements and intervene.

Predictive technologies like early warning systems might avoid failing during learning [15, 16]. An alert works as a strong sign and shows the need to use support and intervention offers [17]. In the USA, universities need to focus on successful students because they increase the reputation and assure funding. In this regard, LA is a powerful tool to identify those students who might fail and to support students in achieving their learning goals [18].

### Learning Analytics in Germany

To use LA in schools and universities, the aspects of pedagogy, complexity, ethics, power, regulation, validity, and affect need to be considered [13]. These aspects are highly dependent on the cultural framework. In Germany, individuality, competition, performance, and success are important cultural factors [19]. Education has a high impact on later opportunities and careers.

Our study is motivated by the ongoing digitization, promoted by the government, and facilitated by the COVID-19 pandemic in Germany. Schools and universities are expansively using platforms to support the learning processes and remote learning [20]; however, these platforms are predominantly used for supplying materials and tests or exams. Due to the General Data Protection Regulation (GDPR) in the European Union and the traditional attitude toward digital technologies, automatic recommendations by LA are unusual. Hence, instructors are not using all the provided functions of platforms that are already implemented. Nevertheless, future developments and the COVID-19 pandemic will change the usage of digital learning systems in Germany.

### Influence on Instructors’ Judgment

Instructors have to evaluate learners’ abilities and competencies, but the precision and correctness of these judgments is sometimes unknown [21]. In traditional education, systematic biases and influences on judgment accuracy are well-studied [11, 22–24]. Biases lead to the problem of unfair grading in school and university contexts. There is evidence that instructors are biased by several personally conditioned factors, such as judgment characteristics and test characteristics, which in turn influence the accuracy [25].

Learning platforms provide new information that can be used for learners’ assessment and can complement the face-to-face sessions [26]. Additionally, LA offers data and analyses about learners and provides insight for the educators, students, and other stakeholders [27]. Hence, recommendations about learners’ success are additional factors when taking the influence of learning platforms on instructors into consideration. To find out how instructors react to the prediction of platforms, we designed a conjoint experiment that offers different kinds of information about the learners.

## Study 1

### Method

Our study uses conjoint analysis that has been applied in numerous judgment and decision-making studies among various disciplines [28]. This methodological approach has several advantages concerning challenges associated with the research context. Researchers can stimulate the participant's decision process in real-time, which is more suitable for spontaneous decision-making, because post-hoc methods are influenced by the participant's tendency to rationalize the decision in retrospective [29, 30]. Adaptive choice-based conjoint analysis is an experimental design and makes causal inference a realistic goal. The adaptive choice-based method is particularly suited to our research question since it produces a decision context that is close to the day-to-day decision context of instructors. Both the experiment and the daily job of participants require a judgment based on a set of observable characteristics.

Participants are asked to judge a series of profiles, which are combinations of parameter values for several attributes. The preferences revealed in this way show the contribution of each attribute’s parameter values to the overall valuation a certain profile receives [29]. Previous research provides substantial evidence for the external validity of conjoint studies [31, 32]. We specifically conducted an adaptive choice-based conjoint experiment since adaptive choice-based conjoint experiments, in contrast to traditional conjoint analysis, come close to the real-life situation of instructors. In general, ACBC choice tasks of selecting alternatives require low cognitive effort [33]. All aspects help to increase both the validity and response rate of the study. The application of this research method to our study is presented in the following paragraphs. An important trade-off in designing an ACBC is making the experiment as realistic as possible while ensuring that it is manageable for respondents. Hence, we decided to restrict each scenario to two students with a maximum of five attributes. Consequently, we selected five attributes based on the research question, we aimed to answer. The design of the experiment is such that all student attributes that do not explicitly vary are equal. Thus, provided the experiment is carefully conducted, the omitted variables do not affect the results.

### Sample

The targeted sample for our online survey were 372 instructors in Germany in the summer of 2020. The mean age was 45 years. 66 percent of the instructors were female and 33 percent male, one respondent was other gender. They all work professionally in educational contexts. The average number of years in the education system was 16 years. 60 percent of the participants have already gained experience with a digital learning platform.

### Experimental Design and Attributes

Prior to the empirical examination, we pretested the experiment with 15 participants to obtain feedback and refine the survey design. The pre-test led us to change the wording of the attribute levels and the introduction to make them more familiar and understandable for instructors. The participants of the pre-test confirmed that the number of choice tasks was indeed manageable, realistic, and understandable.

Participants accessed the experiment online. First, participants were asked to read the text thoroughly and imagine themselves in the described situations (see Appendix). The participants were supposed to give grades to their students at the end of the school year. We chose a grading situation because it reflects a common situation in everyday school life.

In 16 rounds, the instructors were shown the fictitious profiles of two learners with different attributes. They had to choose the one they estimated to be the better performer. The attributes were the given name, the learning behaviour, the number of completed online exams, the extent of parental support, the learner’s picture, and the automatic recommendation by the platform. Each attribute was associated with different levels (Table 1).

**Table 1:** Learners’ attributes and attribute levels

Name	Maximilian, Mohammed, Sophie, Layla
Picture	Generated by AI
Learning behaviour	Activity: never, before an exam, permanent
Exams taken	3/18, 9/18, 17/18
Parental support	Little, moderate, high
Automatic recommendation	Promotion is recommended, Promotion is endangered

Two female and two male learners at the age of about 12 years were the students to be evaluated. Their pictures have been generated by an AI to ensure data protection. The pictures look very pleasing to reduce perception errors that occur through physiognomy [34, 35]. Each image has a name that has not been exchanged to prevent a male image from appearing with a female name and vice versa. To represent different cultures, the given names were typically German and Turkish, because the Turkish minority is the larges in Germany. The attribute learning behaviour was shown as a curve, representing the time spent on the platform. The curves showed low activity, a high activity before an exam, and permanent high activity. Information about passed exams was just demonstrated by the absolute number (3, 9, or 17 of a maximum 18 exams), but no information about the level of difficulty or the content was given. There were three levels of parental support (little, moderate, high). This attribute represents additional exercises at home and support with homework. There is little evidence that parents begin to support primary school pupils when problems arise [36]. Therefore, parental support can be interesting for instructors. The automatic recommendation was expressed with “Promotion is recommended” and “Promotion is endangered”. No information on how the algorithm generated the recommendation was provided. This means the participants did not know which attributes had been rated by the underlying algorithm.

### Results

With the participants’ different preferences, we analyzed which information about learners had the highest impact on the choice. Using the sawtooth software on this ACBC design, the dominance of a few attributes occurred. The exact results are shown in Table 2. First, the participants showed the strongest reaction to the passed exams (32.56 percent of total variability). The more exams a learner had done, the better was the participant’s judgment. Consequently, high activity on the platform and the motivation to take optional exams had a strong effect on the instructors.

**Table 2:** Relative importance of learner’s attributes

Attributes	*R*	*I*
Passed exams	1	32.56 [12.11]
Platform’s recommendation	2	26.32 [13.14]
Learning behaviour	3	20.73 [9.10]
Parental support	4	12.48 [9.82]
Name and picture	5	7.91 [6.59]

*R* rank of each attribute’s importance, *I* relative importance of each attribute (percentage of the total variability)

Second, the participants relied on the platform’s recommendation. They were highly affected by the label “Promotion is recommended” (26.32 percent of total variability). Furthermore, a positive recommendation led to a positive appraisal.

Third, there is little evidence that the participants preferred low parental support. For instance, learners with high parental support were devalued and disadvantaged. Ethnicity, represented by typical names, had a low impact on the participants’ judgment. Likewise, learning behaviour and gender had a neutral effect on the participants.

Attributes are organized according to their importance. *R* is the rank of each attribute’s importance, and *I* is the relative importance of each attribute. It is expressed as percentage of the total variability (high to low) across utility coefficients. Importance scores add to 100.00. The standard deviation is shown in the brackets.

Beyond that, it is important to differentiate between the attribute levels to gain a deeper understanding of the instructors’ preferences (Table 3). The attribute of passed exams had a strong influence with a small and a high number (mean − 75.70 and 75.28). The automatic recommendation had a strong impact (mean − 57.56 and 57.56). The same pattern as the passed exams had the learning behaviour and the parental support. There was a low impact of the level “before an exam” and higher impacts of “never” and “permanent” resp. a low impact of “moderate” and higher impacts of “little” and “high”. There is little evidence that the typical German names had little negative impacts.

**Table 3:** Adaptive choice-based conjoint utility descriptive statistics

Attributes and levels	*M*	SD
Passed exams		
3/18	− 75.70	41.10
9/18	0.42	14.46
17/18	75.28	44.62
Platform’s recommendation		
Promotion is endangered	− 57.56	45.81
Promotion is recommended	57.56	45.81
Learning behaviour		
Never	− 42.74	34.12
Before an exam	− 1.04	16.46
Permanent	43.77	37.84
Parental support		
Little	18.99	35.28
Moderate	2.89	14.97
High	− 21.88	32.10
Name and picture		
Maximilian	− 5.30	19.65
Mohammed	0.248	19.47
Sophie	− 0.85	19.78
Layla	5.90	19.65

*M* mean, *SD* standard deviation

## Study 2

### Research Question

Study 1 revealed the importance of exams and recommendations on instructors’ judgment. However, the question remains whether the influence is the same for every learner and to what extent new information of a learning management system (LMS) influences the judgment. In Study 1, we provided no traditional information about the learners, such as classwork, participation, or presentation. A LMS can provide insights into the online learning behaviour of students [37], but knowledge is scarce whether and how instructors consider this information for grading. There might be differences in considering information or LA recommendation from LMS platforms. We created a within-subject experiment that reflects the traditional grading situation at the end of the school year combined with additional data and a recommendation from an LMS.

### Study Design

We designed a two-stage scenario-based between-subject experiment [38] to examine how instructors react to the additional insights about the learning process and which personal differences occur. In a first step, we asked participants to imagine the situation that they must grade four fictive students based on their personal notes and the grades the students achieved during the year on a traditional German scale (see Appendix, Table 6). The description of the four students did not include different names due to the small effect discovered in our Study 1.

In a second step, we added data from the learners’ digital behaviour on the learning platform. Participants received the report by the LMS about the four students, which shows the time spent on platform, the tests completed, an overview of the grades (corresponding to the information of the first step), and the platform’s recommendation whether the student is at risk or has a good learning prognosis (see Appendix, Table 7). We asked participants to judge the same four students again. We designed the graphical output of the platform in the style of existing platform currently used in schools. Moreover, we gave no additional information about how the platform’s recommendation was calculated to address the opacity of an automated system. The output showed a recommendation just for two learners to control for the impact of a prognosis on different kinds of learners and their learning behaviour.

The two measurement points allow us to compare the different judgments for each student. Additionally, we were able to compare the similarity of the judgments regarding whether the information provided by the LMS changed the individual grades or not. The instructors had to evaluate after the two steps on a 7-point Likert scale how confident they were with their grading and if they have taken the LMS’s information into account (information about activity, completed exams and recommendation).

### Sample

We recruited 100 instructors from Germany using an online panel of an ISO 20252:2019 certified online sample provider in summer 2021. After excluding five participants due to unrealistic answers (i.e., no differences in grading between the learners or answers out of the possible range), our final sample was 95 participants. On average, the instructors were 44.44 years old [SD = 11.27] and had 15.52 [11.34] years of work experience. 67 percent were female. Due to their job title, they were all familiar with the German grading system.

### Results

With the comparison of two measurement points, our study reveals first important insight to the impact of LMS data and recommendations. Table 4 shows the differences between the two steps. In Germany, grades are generally based on the numbers 1–6, where 1 is the best and 6 the worst grade. It is common, to use tendencies with plus and minus between these numbers to show that a student is slightly better or worse. We used the whole scale with plus and minus, so the results are based on a scale from 1 to 16, with a 1 for the best grade and 16 for the worst grade.

**Table 4:** Change in the individual grading

Learner	Step 1 (Traditional information)		Step 2 (+ LMS information)	
	Grade mean [SD]	Perceived confidence mean [SD]	Grade mean [SD]	Perceived confidence mean [SD]
A	10.86 [0.88]	5.32 [0.97]	10.17 [1.17]	5.32 [0.84]
B	8.78 [1.05]	5.26 [0.91]	9.12 [1.15]	5.24 [0.93]
C	5.35 [1.00]	5.11 [0.87]	5.64 [1.18]	5.08 [0.91]
D	3.02 [0.60]	5.48 [0.90]	2.77 [0.68]	5.58 [0.82]

Note: 1 stands for the best possible grade, 16 stands for the worst possible grade. For example, a mean of 11 represents a grade of 4—in the German system, while a mean of 10 represents a 4

The descriptive statistics show that there are differences between step 1 and step 2. This might be a first hint for an impact of the additional information, which must be specified in the further analysis. Overall, the perceived confidence by participants with their grading was moderate to high and showed almost no differences.

The differences can have various reasons. Therefore, our analysis has an explorative character. In the following, we describe the effects of characteristics that can be important to explain the differences. To find out, how the differences between step 1 and step 2 can be explained, we calculated new variables “Difference from Step 1 to Step 2” and “Change from Step 1 to Step 2” for all learners. We wanted to explain to which extend instructors change their judgment and sought to control our finding with the dummy variable with a 1 for a changed judgment. We also calculated a new variable which shows the difference in perceived confidence.

Running a hierarchical regression using the R statistical software for each learner and for both dependent variables, the effects of high and low activity, the recommendation, and the change in confidence are depicted in Table 5. Instructors reacted positive to high activity, which is evident for Learner A and D. The recommendation was important when it was a warning. The instructors reacted negative to the LMS’s recommendation for Learner B, and this negative impact was significant. A positive recommendation seemed to confirm what the instructors already knew. All in all, activity, and recommendations on the LMS are important information for instructors. A high activity can lead to better judgment for high and low achievers in the classroom.

**Table 5:** Regression analysis of the differences (Study 2)

Coefficients	Differences Step 1 to Step 2				Change Step 1 to Step 2 (Dummy)			
	Estimate	SE	*t* value	*Pr* ( >|*t*|)	Estimate	SE	*t* value	*Pr* ( >|*t*|)
Learner A								
(Intercept)	− 0.52	0.33	− 1.60	0.11	− 0.13	0.17	− 0.76	0.45
Work exp	0.00	0.01	0.48	0.63	− 0.00	0.00	− 0.94	0.35
Activity LMS	0.26	0.07	3.90	0.00**	0.17	0.03	4.83	0.00**
Rec. LMS	− 0.03	0.06	− 0.49	0.63	− 0.02	0.03	− 0.56	0.58
Confidence	− 0.30	0.15	− 2.02	0.05*	− 0.12	0.08	− 1.52	0.13
	Multiple *R*-squared: 0.19, Adjusted *R*-squared: 0.15 *F*-statistic: 5.25 on 4 and 90 DF, *p* value: < 0.01				Multiple *R*-squared: 0.25, Adjusted *R*-squared: 0.21 *F*-statistic: 7.40 on 4 and 90 DF, *p* value: < 0.01			
Learner B								
(Intercept)	− 0.14	0.28	− 0.48	0.63	− 0.01	0.17	− 0.06	0.95
Work exp	0.01	0.01	0.69	0.50	− 0.00	0.00	− 0.49	0.63
Activity LMS	0.05	0.06	0.76	0.45	0.01	0.04	0.26	0.80
Rec. LMS	− 0.13	0.05	− 2.57	0.01*	0.08	0.03	2.67	0.01*
Confidence	− 0.39	0.21	− 1.92	0.06	0.07	0.12	0.57	0.57
	Multiple *R*-squared: 0.10, Adjusted *R*-squared: 0.06 *F*-statistic: 2.44 on 4 and 90 DF, *p* value: 0.05				Multiple *R*-squared: 0.10, Adjusted *R*-squared: 0.06 *F*-statistic: 2.60 on 4 and 90 DF, *p* value: 0.04			
Learner C								
(Intercept)	0.09	0.24	0.39	0.70	− 0.14	0.17	− 0.85	0.40
Work exp	0.00	0.01	0.53	0.60	− 0.00	0.00	− 0.24	0.81
Activity LMS	− 0.09	0.05	− 1.77	0.08 +	0.10	0.03	2.85	0.01*
Rec. LMS	− 0.00	0.04	− 0.11	0.92	− 0.00	0.03	− 0.12	0.90
Confidence	− 0.13	0.12	− 1.04	0.30	0.09	0.08	1.13	0.26
	Multiple *R*-squared: 0.06, Adjusted *R*-squared: 0.02 *F*-statistic: 1.4 on 4 and 90 DF, *p* value: 0.23				Multiple *R*-squared: 0.11, Adjusted *R*-squared: 0.07 *F*-statistic: 2.85 on 4 and 90 DF, *p* value: 0.03			
Learner D								
(Intercept)	− 0.26	0.16	− 1.60	0.11	− 0.24	0.15	− 1.60	0.11
Work exp	− 0.00	0.00	− 0.69	0.49	− 0.00	0.00	− 0.70	0.49
Activity LMS	0.09	0.03	2.75	0.01*	0.09	0.03	2.82	0.01*
Rec. LMS	0.03	0.03	0.96	0.34	0.03	0.03	0.94	0.35
Confidence	0.07	0.09	0.73	0.47	0.07	0.08	0.86	0.39
	Multiple *R*-squared: 0.15, Adjusted *R*-squared: 0.11 *F*-statistic: 3.99 on 4 and 90 DF, *p* value: 0.01				Multiple *R*-squared: 0.16, Adjusted *R*-squared: 0.12 *F*-statistic: 4.18 on 4 and 90 DF, *p* value: 0.01			

Notes: Variables were measured as follows:

Work exp. = Instructor’s work experience in years

Activity LMS = Importance of the information on learner’s activity (7-point Likert scale; 1 = not important, 7 = very important)

Rec. LMS = Importance of the information on recommendation (Learner B: at risk, Learner D: good prognosis; 7-point Likert scale, 1 = not important, 7 = very important)

Confidence: Instructor’s confidence with his/her decision (7-point Likert scale; 1 = not confident, 7 = very confident)

Signif. codes: + *p* < 0.1 *p < 0.05; **p < 0.01

## Discussion

These studies aimed to examine the influence of LA’s recommendations on instructors’ judgment in the educational context and the influence of information about the behaviour online. Besides the number of passed exams, results showed that instructors heavily rely on LA’s recommendation about the promotion of a learner to the next grade as well as her/his depicted learning behaviour. Parental support and the name with the picture of the learner had only little influence on instructors.

The high degree of influence by LA’s recommendations is surprising because participants in our study had no additional information about how the LA system was trained, how the system predicted the learning success or what information was used to make this recommendation. Although one might assume higher objectivity in assessing and evaluating learning outcomes by a computer system rather than a human, the literature discussed the problems of potential biases and discrimination of machine learning systems [39]. Besides the LA recommendation, learning behaviour ranked third in the relative importance for instructors to evaluate learners. LA systems cannot analyze offline-learning activities, which can lead to a disadvantage for offline learners and biases in judgment [40]. This results in implications for theory and practice.

### Theoretical Implications

Using algorithms in learning contexts can be useful to generate deeper insights into the learning processes [41]. But algorithms’ accuracy is highly dependent on the training data, and the results are not comprehensible. This leads to the problem of opacity when using algorithms. Opacity means that users get a result without knowing the relationship between data and the algorithm [42]. But taking the platform’s recommendation without giving it serious consideration can over- and underestimate a learner’s learning success. Consequently, learners do not get the right support, or their learning performance is rated too low. Leaving all the decisions to the platform means a high risk of unfair judgment [43]. Therefore, further research is needed to understand how traditional judgment and LMS and LA systems can work together in a meaningful way. The level of activity is an important sign of effort and can reveal information about the learning process that was hidden in traditional contexts.

There is a need for transparency when using algorithms for decision-making. This means users should be informed about the data which are used for decisions. Adding transparency to algorithms is difficult because high transparency complicates the use and can encourage misuse of the system [44]. Nevertheless, auditing of systems is necessary, and suitable concepts will be developed with increasing use.

### Practical Implications

Instructors play an essential role in educational outcomes [45]. Although learning is a sensitive process, instructors are impacted by numerous personally conditioned factors, e.g. from self-fulfilling prophecies [9]. Relying on valid and observable indicators can improve judment accuracy, like Urhahne and Wijnia [11] advise. The results of LA systems seem to be these valid and observable indicators and therefore, the context in which the results are used becomes relevant. Specific patterns in the learner’s online behaviour can be integrated into an early warning system to ensure that their learning success is endangered. If the algorithmic decision is used for judgment, the aspects of equal opportunities must be taken into consideration. Algorithms can support decision-making, but the outcome can be biased depending on the training data and the chosen model [46].

To understand the operations of platforms, it is necessary to know how algorithms work and predict certain outcomes. Therefore, educational institutions need to develop the instructors’ knowledge and train their digital competencies about LA systems and algorithms [47] because a limited understanding of these new technologies in combination with little experience will lead to unwanted effects, such as reproducing stereotypes, biases, and discrimination. There are ongoing processes to develop measurable concepts like AI literacy [48] that represent the basic skills and abilities. If instructors are aware of these emerging problems, platforms can create learning success through better internal differentiation in the classroom and focus on the specific problems revealed by data.

The second study revealed a positive impact of high activity. On the one hand, this can be a change for very quiet and shy learners. Maybe they are underestimated when instructors get to know them in the classroom. Having an impression of their learning behaviour can support a positive image and for this reason better grades. On the other hand, a low activity can have different reasons. Some learners might prefer offline learning and can be very successful with their strategy. For others, a low activity might be a warning signal for occurring problems. In this case, LMS data can lead to earlier interventions and help the learners. All in all, there is no general recommendation for using LMS data, but personal learning strategies must be considered.

### Limitations and Future Research

First, the choice experiment approach brings unique advantages for studying decision criteria, but it comes with caveats. Conjoint analysis research reduces the social desirability and retrospective reporting biases associated with self-reports of judgments. Judgments are made in a relatively controlled environment. But one cannot be sure that participants were mentally able to keep all other start-up attributes equally. These limitations are true for all choice experiments, and we have paid particular attention to designing the experiment as realistically as possible to alleviate these concerns. Although we selected the most essential attributes identified by previous literature, the choice experiment approach implies that we can study only a limited set of start-up attributes. Importantly enough, this feature does not affect the results.

Second, the tested setting assumed that the instructors evaluated the learners only based on the information provided by the platform. In everyday school life, however, it is more conceivable that the platform could be used to support the learning processes. Therefore, instructors at school would supplement their own impression of the learners with the information rather than relying solely on it. The situation at universities is different. There is usually a less strong personal relationship between lecturers and students due to the high number of students. This means that the use of learning platforms can have a different impact in the university context, which is more similar to the first study than schools with smaller classes.

Third, different current social discourses may influence the result, for instance, the reactions to the Black Lives Matter movement since May 2020. Maybe, our participants were aware that learners and students of color are often discriminated against in educational contexts. This might explain the positive impact for Turkish names, but further research is needed to explain these differences because minorities can be discriminated against. For instance, there is evidence for the underrepresentation of students of color in gifted programs in the USA [49].

Forth, the second study has an explorative character due to the combination of traditional and LMS data. The sample is small so the effects cannot be generalized. Maybe, other effects of personal characteristics are still hidden.

Finally, our research was conducted in only one country (Germany). Thus, the question of cross-national generalization remains open due to different school and university systems, different levels of digitization of educational institutions and cultural differences [19]. Future research, therefore, should be conducted in different cultures to fully assess generalization.

## Conclusions

We sought to increase the current understanding of LA algorithms in educational contexts. Driven by the current challenges due to the COVID-19 pandemic, teaching routines in schools and universities may change, and so may the impact of platforms. Our work showed that instructors heavily relied on the recommendations by the LA system when no other information is provided. The use of platforms enables instructors to get access to hidden patterns of learning behaviour. In addition to the traditional classwork, they combine the new information with traditional grades to get a better impression. For practice, these insights provide a better allocation of personal support. Furthermore, using LA tools implies focusing on the learner's activities on platforms. There might be other relevant activities, which are meaningful for learning success but are not represented in analytics [40]. Further research is also needed to find out, which learners benefit most from information about their learning behaviour and how LMS can be designed to provide the important information for both instructors and learners. There are opportunities for deeper insights to the individual learning habits. Instructors can understand more precisely when and which learning problems occur and can intervene to support their learners.

## Figures and Tables

**Table 7:**
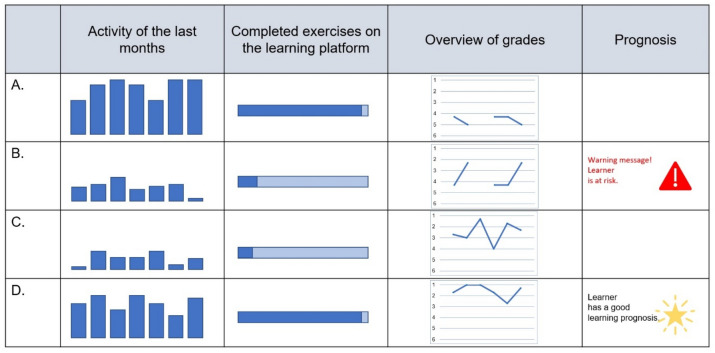
Given LMS output (Study 2)

## Data Availability

Upon reasonable request.

## Description of the Situation (Study 1): Introduction for the participants

The school year is coming to an end, and the summer vacations are approaching. In a few days, you will have to enter the grades for your 10th class consisting of 32 students to write the reports afterward.

For grading purposes, the school's internal learning platform provides you with the name, a picture and the type of learning type of each student. A distinction is made between three different types. The learning type “not at all” describes students who do not repeat the school material independently and do not prepare for exams. They hardly or not at all use the school's internal learning platform. Students who are “permanently” learning to learn the relevant content regularly throughout the school year and actively use the school's internal learning platform for this purpose. The learning type “always before exams” refers to students who learn only in a short period before exams or exams or who use the school's internal learning platform. In the remaining time of the school year, they have a low learning activity.

Furthermore, you know to what extent parents support their children in terms of school success. A distinction is made between no, moderate and much support from the parents. Parents who provide a lot of support are informed about the subjects, contents, and current school events. They regularly talk to their children about these topics and help with any problems the children may have with the content or social issues. In contrast, parents who do not provide support have little knowledge of their children's school situation and development. They do not support their children in case of content-related or social difficulties. Moderate support from parents corresponds to an occasional commitment. The parents are informed about the general situation at school and help in major difficulties with the content or social problems.

You can also see which learners have been classified as “at-risk” by the digital learning platform. According to the platform, those students are at risk of not being transferred. Indicators for such a threat are the extent of reading activity, adherence to due dates, participation in forums and written submissions.

In the following, you will be presented 16 times with two students, each with the above information, and you will be asked to choose which one you rate better. Afterward, you will be asked some more questions.

See Tables 6, 7.

**Table 6:** Given information about the learners (Study 2)

Learner	Notes	Classwork 1	Participation	Voluntary Presentation Leistung	Test	Classwork 2	Participation
A	A. is very quiet, often absent, has little contact with classmates and seems very withdrawn	4	5	–	4-	4-	5
B	B. is outgoing, rather restless and likes to talk to friends	4 −	3 +	–	4	5	2
C	C. stands out in class by asking questions frequently and wants to get good grades	3 +	3	1 −	4	2 +	2-
D	D. is active and hardworking and has a large circle of friends at school	2	2 +	1	2 −	1	2 +

Note: In Germany, the 1 is the best grade while a 6 is worst. Plus and minus show trends.

## Code Availability (Software Application or Custom Code)

Upon reasonable request.
